# Comparative analysis of paraffin and JB-4 embedding techniques in light microscopy

**DOI:** 10.1093/biomethods/bpaf071

**Published:** 2025-11-07

**Authors:** Zeynep Deniz Şahin İnan, Rasim Hamutoğlu, Serpil Ünver Saraydın

**Affiliations:** Department of Histology and Embryology, Faculty of Medicine, Sivas-Cumhuriyet University, Sivas 58140, Turkey; Department of Histology and Embryology, Faculty of Medicine, Sivas-Cumhuriyet University, Sivas 58140, Turkey; Department of Histology and Embryology, Faculty of Medicine, Sivas-Cumhuriyet University, Sivas 58140, Turkey

**Keywords:** JB-4 technique, liver, long bone, morphology, paraffin technique

## Abstract

Histological embedding and staining techniques are essential for examining tissue and cellular morphology. This study compares two embedding methods—JB-4™, a glycol methacrylate-based resin, and conventional paraffin—to determine which method provides superior visualization of liver and long bone tissues under light microscopy. Liver tissues from both embedding protocols were stained using the Periodic Acid–Schiff method and silver impregnation method. JB-4 sections were also stained with acid fuchsin and toluidine blue, while paraffin sections were stained with hematoxylin and eosin staining. Contrary to the common assumption that JB-4 may interferes with certain staining protocols, acid fuchsin and toluidine blue yielded high-contrast, structurally detailed results in JB-4 sections. Both techniques preserved liver morphology. However, JB-4 demonstrated higher resolution and enhanced visualization of intracellular structures. JB4 also preservedglycogen more effectively. Cellular structures including nuclei, nucleoli, bile duct epithelial cells, and Kupffer cells, were observedmore distinctly in JB-4 preparations. Reticular fibers were similarly visualized with both embedding techniques. In contrast, paraffin embedding provided better preserved overall tissue architecture. Whilelong bone specimens, paraffin sections frequently displayed poorly defined structures, while JB-4 offered clearer visualization of chondrocyte lacunae, osteocyte nuclei, lamellar bone, and bone marrow cells. JB-4 and paraffin each offer distinct advantages depending on tissue type and histological objective. JB-4 appears to be compatible with a broader range of stains than was previously reported, which expands its utility in detailed tissue analysis. The selection of an embedding method should align with the morphological characteristics of the target tissue and the specific research goals.

## Introduction

In histology, various methods are employed for examination under light microscopy, each presenting specific advantages and limitations. Despite the widespread use of paraffin, alternative embedding techniques like JB-4, though less common today, offer distinct advantages that warrant reconsideration, especially in terms of staining compatibility and cellular resolution. Accurate interpretation of the cytological features requires that specimens be preserved as close to their native state as possible throughout the preparation process. During this stage, the embedding medium should be practical, easy to apply, cost-effective, and capable of providing detailed visualization for microscopic evaluation.

Paraffin embedding has long been the most widely used technique for light microscopy. Introduced to histological practice by Edwin Klebs [1], paraffin enables sufficient tissue hardening for thin sectioning suitable for microscopic analysis. A range of paraffin melting points facilitates optimal sectioning across various tissue types. Among its advantages, paraffin embedding preserves tissue morphology over extended periods and is compatible with a wide range of staining techniques, including immunohistochemistry and *in situ* hybridization. However, paraffin also presents limitations, including low thermal conductivity, volumetric changes during phase transitions, flammability, and a tendency to render tissues brittle when overheated. Furthermore, obtaining ultra-thin or semi-thin sections for enhanced cellular resolution is challenging with paraffin.

To overcome these limitations and improve cellular detail preservation, the JB-4 embedding technique was developed and introduced in the 1970s [2]. This method utilizes a water-soluble plastic resin, glycol methacrylate, also known as 2-hydroxyethyl methacrylate (HEMA). Unlike paraffin embedding, JB-4 does not require toxic clearing agents such as xylene or chloroform, thereby offering a more user-friendly and environmentally safer protocol. While JB-4 has been traditionally considered incompatible with certain special stains, our findings challenge this view by demonstrating its effective compatibility with acid fuchsin and toluidine blue, expanding its applicability beyond conventional expectations. JB-4 enables production of ultra-thin (0.5–1 µm) and semi-thin (2–3 µm) sections, allowing superior cytological detail. Additionally, polymerization occurs at room temperature or 4°C, and the resulting resin is harder than paraffin, facilitating thinner sections with fewer artifacts [2]. JB-4 is especially suitable for small or undecalcified bone specimens and compatible with various histological stains. Nevertheless, concerns remain regarding the inability to remove glycol methacrylate from sections and its potential to obscure antigenic sites, as noted in some studies [3].

This study systematically compares the effectiveness of paraffin and JB-4 embedding techniques for the light microscopic examination of liver and bone tissues. Emphasizing JB-4’s staining versatility and superior morphological preservation, our aim is to revitalize interest in JB-4 embedding for detailed histological analysis, especially in tissues requiring enhanced cytological resolution. We employed routine, PAS, and silver staining methods to to evaluate the structural and cytological differences between the two techniques.

## Materials and methods

### Experimental animals

This study utilized five adult female *Wistar* albino rats, aged four months and weighing approximately 250–300 grams. Sourced from a local experimental animal facility, the animals were housed under controlled conditions. Housed at room temperature under a 12-h light/dark cycle, the rats provided standard pellet feed and tap water *ad libitum* until tissue collection. Ethical approval was granted by the local Animal Experiments Ethics Committee (Approval No: 65202830-050.04.04.-13, dated 05.03.2025).

### Tissue preparation

Euthanasia was performed *via* intraperitoneal injection of sodium pentobarbital (30–50 mg/kg) (P3761, Sigma-Aldrich, USA) prior to collecting liver and bone tissue collection. Tissues intended for paraffin embedding were fixed in 10% neutral buffered formalin (HT501128, Sigma-Aldrich, USA) for 48 h. Tissues destined for the JB-4 embedding were fixed in 3% glutaraldehyde (G5882, Sigma-Aldrich, USA). Bone tissues were decalcified using a rapid decalcification solution (BS-064, BesLab, Türkiye), while liver specimens underwent routine tissue processing.

In this study, our primary objective was to compare the embedding media themselves rather than staining variability. JB-4, a glycol methacrylate–based hydrophilic resin, enables polymerization without post-sectioning dehydration and xylene clearing and is conventionally visualized using Acid Fuchsin and Toluidine Blue [4]. In contrast, paraffin, a hydrocarbon-based hydrophobic medium, requires graded alcohol dehydration and xylene clearing prior to infiltration and is routinely coupled with hematoxylin and eosin staining [5]. Accordingly, the different staining protocols applied in this study reflect the intrinsic chemical properties and established histological practices associated with each embedding medium.

Tissues for paraffin embedding were processed using an automated tissue processor. Samples were dehydrated through graded ethanol concentrations (50%, 70%, 80%, 90%, 96%, and 100%) (459836, Merck, Germany), each step lasting 2.5 h. Following dehydration, tissues were cleared in xylene (two changes, 2 h each) (534056, Sigma-Aldrich, USA), infiltrated with molten paraffin for approximately 8–10 h, and embedded into paraffin blocks.

Fixed and decalcified samples for the JB-4 technique were dehydrated through an ascending ethanol series (70%, 80%, 90%, and 96%) for 1.5 h per step using a tissue processor. This was followed by two incubations in absolute alcohol (100%), each lasting 1 h. Dehydrated tissues were incubated overnight in infiltrating solution, a mixture of JB-4A solution (J4954, Sigma-Aldrich, USA) and catalyst (J0455, Sigma-Aldrich, USA). On the following day, tissues were embedded in glycol methacrylate-based plastic resin—a catalyzed mixture of JB-4A and JB-4B solutions (J0330, Sigma-Aldrich, USA)—and polymerized at 4°C for three days, following Bulut’s procedure (as described in Bulut’s 1996 dissertation) [6].

Sectioning JB-4 embedded tissues differed from paraffin in several aspects; notably JB-4 blocks were cut using glass knives. JB-4 blocks were sectioned at room temperature, and their harder consistency allowed thinner sections without chilling. Due to their fragility, sections were collected individually without ribboning. Before mounting on poly-L-lysine-coated slides, sections were floated on a warm water bath (∼40°C) to minimize wrinkling, then air-dried at room temperature. In contrast, paraffin blocks were sectioned with steel blades, ribboned, floated on a water bath, and dried in an oven.

### Staining methods and imaging

Liver and bone sections (∼3–4 μm thick) were obtained from paraffin blocks using a rotary microtome (Leica, Germany). Sections were stained with hematoxylin and eosin (H&E) (ab245880, Abcam, UK) to assess general morphology. Liver tissues were further stained with Periodic Acid-Schiff (PAS) (BS 040, BesLab, Türkiye) and silver impregnation (BO 04-040801, Bio-Optica Milano, Italy) to highlight glycogen granules and reticular fibers, respectively.

PAS and silver impregnation staining procedures were performed according to the manufacturers’ instructions.

**Figure 1:**
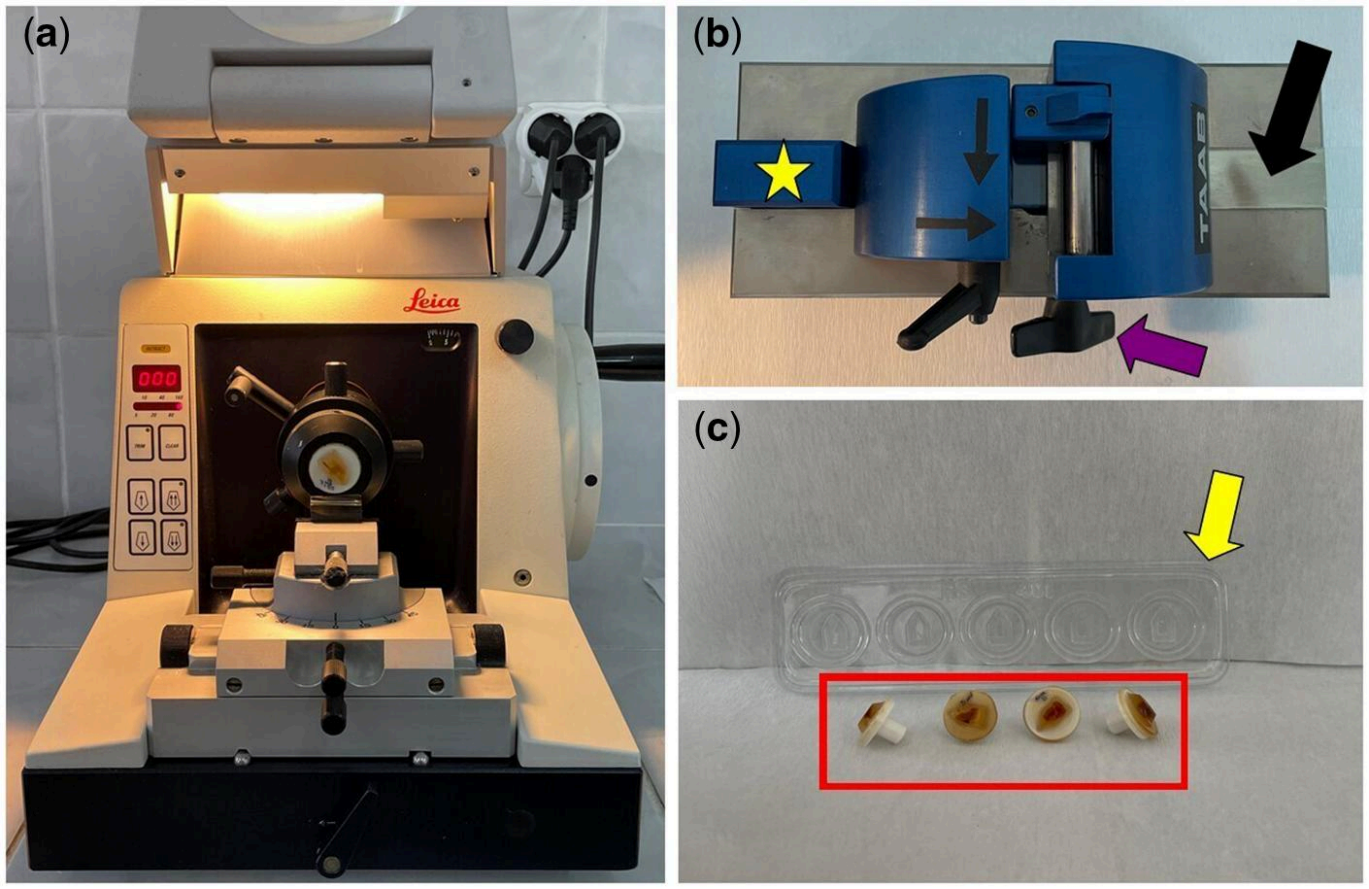
Equipment and materials used for the JB-4 technique. (**a**) Specialized microtome for semi-thin sectioning of JB-4 embedded tissues, showing the block holder and blade holder typical of a microtome but with modifications specific for JB-4 blocks, (**b**) Glass knife maker used to prepare glass knives for JB-4 sectioning. These knives are similar to electron microscopy knives but slightly thinner. The glass blade is placed inside the device, and cutting is performed using the arrows and a lever. The knife is then carefully removed from the star-shaped holder, (**c**) Block holder and JB-4 specific blocks (red square) used during embedding and sectioning (Yellow arrow: Block mold)

For JB-4-embedded samples, semi-thin sections (∼1–1.5 μm thick) were cut using a specialized JB-4 compatible microtome (Leica RM2045, Germany) (Fig. 1a). Glass knives required for sectioning were prepared using a dedicated knife maker (Fig. 1b). During embedding and sectioning, blocks were held in a JB-4-specific block holder (Fig. 1c). General tissue morphology was assessed using Acid Fuchsin (117920, Sigma-Aldrich, USA) and Toluidine Blue (T3260, Sigma-Aldrich, USA) staining. Sections were immersed in 1% Acid Fuchsin for two minutes, rinsed in distilled water for one minute, incubated in 0.05% acetate-buffered Toluidine Blue (pH 4.4) for 2 minutes and rinsed again for 30 seconds, and air-dried. Coverslips were mounted using Entellan (107961, Merck, Germany). Stained sections from both embedding techniques were imaged using an Olympus BX51 microscope (Tokyo, Japan).

## Results

**Figure 2:**
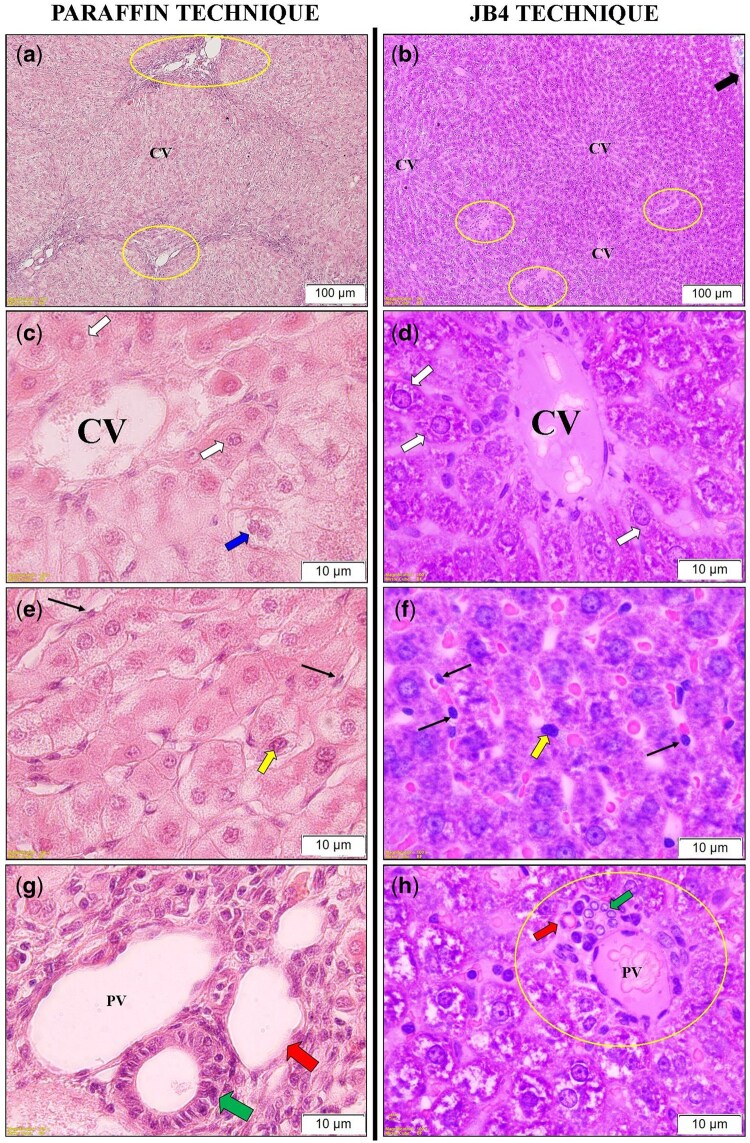
Morphological comparison of liver sections prepared using paraffin and JB-4 embedding techniques. Panels (**a**) and (**b**): General view of the liver parenchyma in paraffin and JB-4 sections at the same magnification, showing the central vein (CV), portal triad (Yellow circled area), and Glisson capsule (thick black arrow). Panels (**c**) and (**d**): Higher magnification views of the central vein and hepatocytes (White arrow) (Blue arrow: Binucleated hepatocyte). Panels (**e**) and (**f**): Kupffer and endothelial cells within the sinusoids were highlighted. Kupffer cells (Yellow arrow) are larger, irregularly shaped, and more darkly stained, whereas endothelial cells (Thin black arrow) are thin, flat, and line the sinusoidal walls. Panels (**g**) and (**h**): Higher magnification images of structures within the portal triad (PV: Portal vein; Red arrow: Hepatic artery; Green arrow: Bile duct). Scale bars: (a) and (b) = 100 µm; (c)–(h)  = 10 µm. Panels (a), (c), (e), and (g) show hematoxylin&eosin (H&E) staining, while panels (b), (d), (f), and (h) show Acid Fuchsin&Toluidine Blue staining

In the H&E-stained liver sections obtained from paraffin-embedded tissues, the overall preservation of tissue morphology was adequate. Hepatocyte cords (Fig. 2c), sinusoids (Fig. 2e), central veins (Fig. 2a and c), and portal triad regions (Fig. 2a and g) surrounding the classical liver lobules were clearly identifiable. In some areas, binucleated hepatocytes were observed (Fig. 2c). Although endothelial cells lining the sinusoids could be distinguished, identification of Kupffer cells remained difficult (Fig. 2c and e).

In contrast, liver tissue sections embedded in glycol methacrylate (JB-4) and stained with acid fuchsin–toluidine blue demonstrated superior morphological preservation and compatibility with the staining protocols employed. High-resolution imaging of JB-4 sections revealed detailed intracellular structures with minimal tissue distortion and shrinkage. JB-4 embedding, known for its superior compatibility with water-soluble components, allowed improved glycogen preservation compared to paraffin embedding, as later demonstrated by PAS staining results. Nuclear and nucleolar structures of hepatocytes appeared with enhanced contrast and detail (Fig. 2d, f, and h). The shape and organization of endothelial cells surrounding the central vein were more clearly delineated (Fig. 2d). Additionally, Kupffer cells within the sinusoids could be readily identified based on their nuclear and cytoplasmic features (Fig. 2f). In the portal triad regions, the cuboidal epithelial cells of the bile ducts displayed well-defined cellular morphology (Fig. 2h).

**Figure 3:**
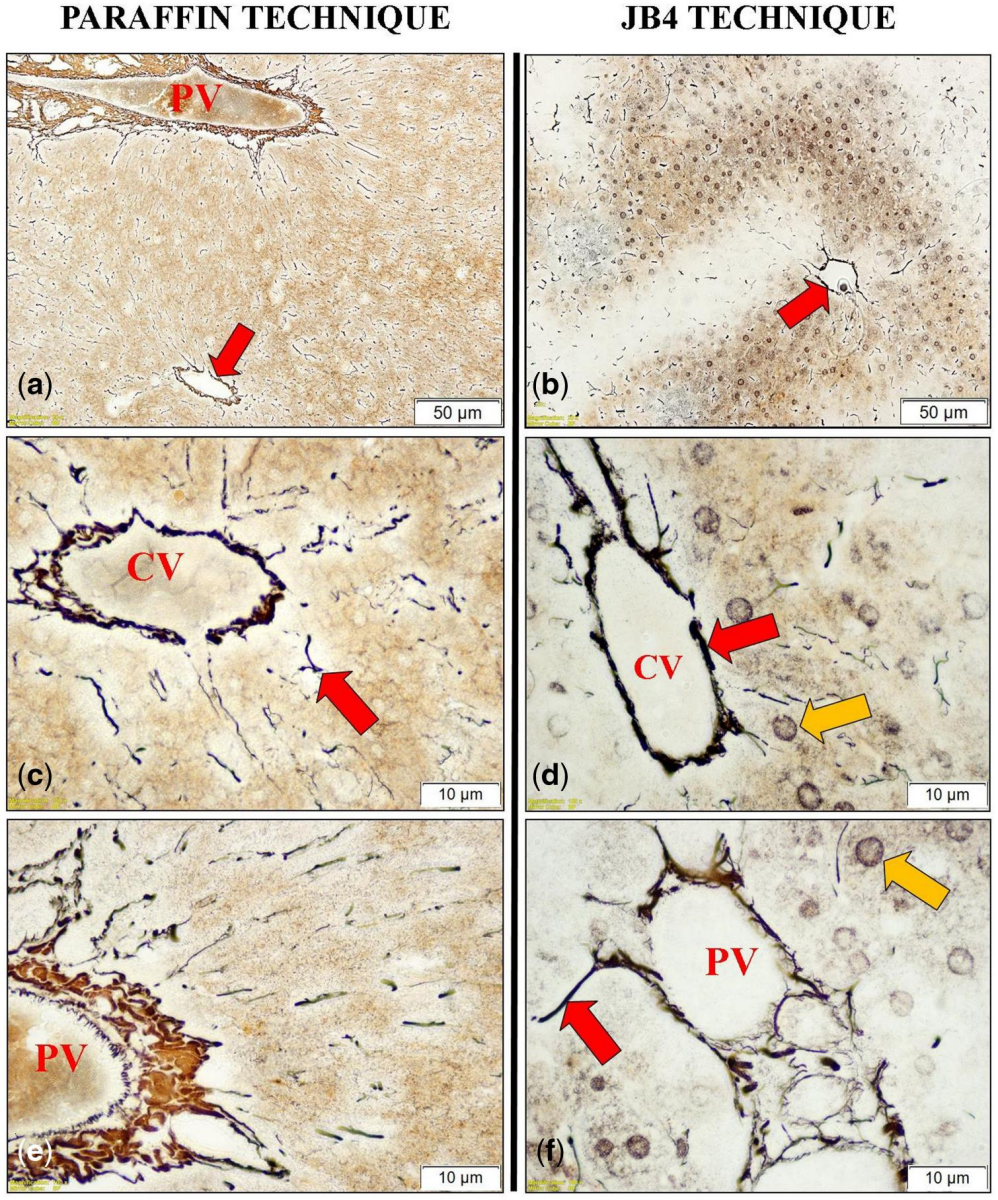
Reticulin staining (performed using silver impregnation) of liver tissue sections processed with paraffin and JB-4 embedding techniques. Panels (**a**) and (**b**): General appearance of reticular fiber distribution in paraffin- and JB-4-embedded liver sections (PV: Portal vein; Red arrow: Reticular fibers). Panels (**c**) and (**d**): Higher magnification images showing reticular fibers located within the connective tissue between hepatocytes (Yellow arrow) and surrounding the central vein (CV). Panels (**e**) and (**f**): High magnification images showing reticular fibers within the connective tissue around the portal triad and between hepatocytes. Scale bars: (a) and (b) = 50 µm; (c)–(f)  = 10 µm

Both the silver impregnation and PAS staining methods produced satisfactory results in liver sections prepared with either embedding technique. Reticular fibers, as revealed by silver staining, were predominantly concentrated around the portal triad and central vein regions (Fig. 3a–f). However, the nuclear details of hepatocytes—clearly visible in JB-4-embedded sections—were poorly preserved or absent in paraffin-embedded counterparts (Fig. 3d and f).

**Figure 4:**
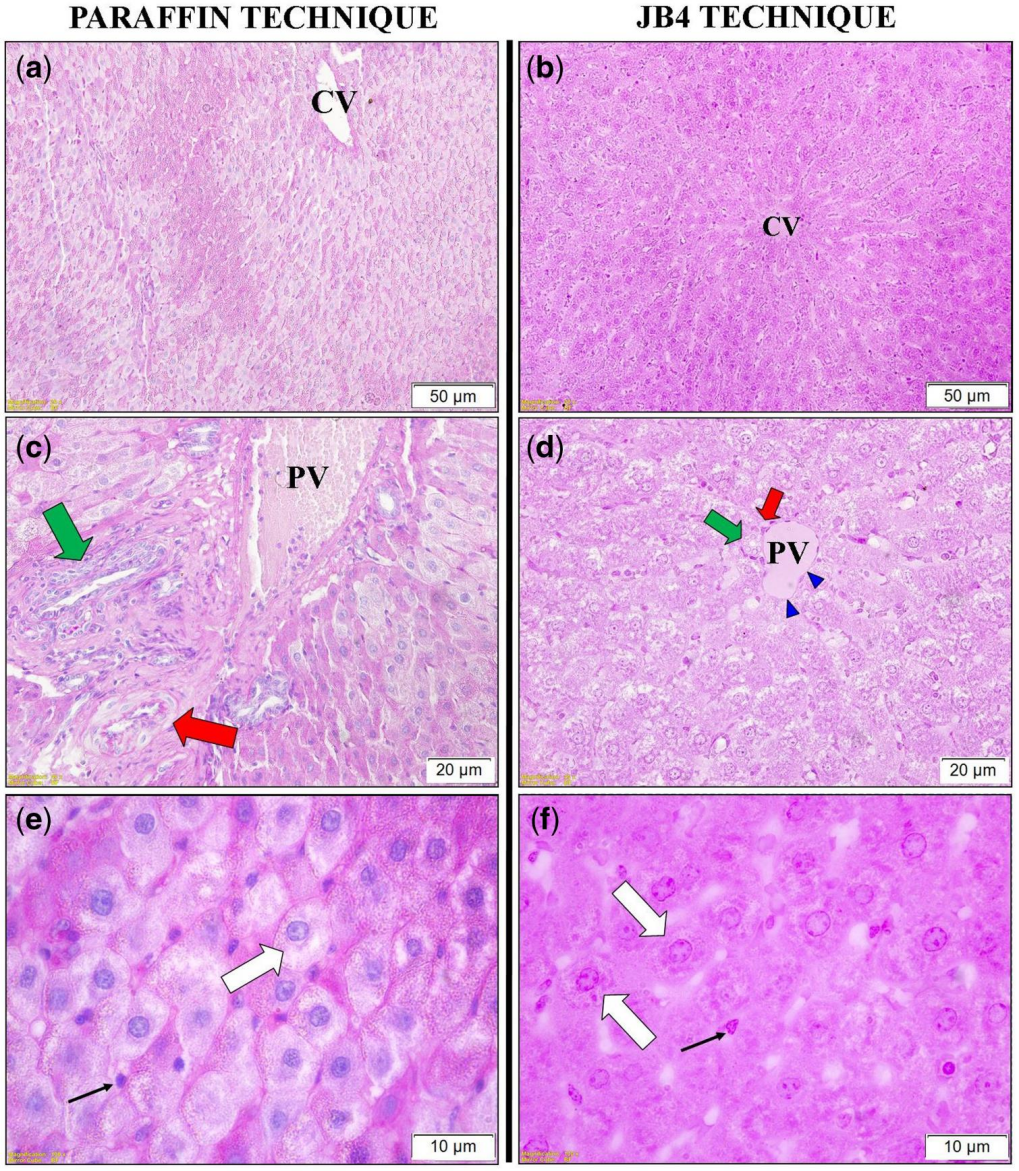
Comparative PAS staining in liver tissue sections prepared by paraffin and JB-4 techniques. Panels (**a**) and (**b**): General view of liver tissue at low magnification showing hepatocytes around the central vein (CV). Panels (**c**) and (**d**): Higher magnification of hepatocytes located around the portal triad, showing cytoplasmic PAS-positive glycogen granules. In panel (d), a basement membrane-like PAS-positive structure (arrowhead) was seen surrounding the portal vein (PV: Portal vein; Red arrow: Hepatic artery; Green arrow: Bile duct). Panels (**e**) and (**f**): Higher magnification (100×) showing glycogen deposits in hepatocytes for comparative analysis. In paraffin sections (e), glycogen appeared as fine, delicate cytoplasmic granules, whereas in JB-4 sections (f), they were often observed as coarser, and more conspicuous aggregates (White arrow: Hepatocytes; Thin black arrow: Endothelial cells). Notably, nuclei in paraffin sections appear blue due to Harris hematoxylin counterstaining, whereas nuclei remain unstained in JB-4 sections, reflecting the different staining behavior of the two embedding media. Scale bars: (a) and (b) = 50 µm; (c) and (d) = 20 µm; (e) and (f) = 10 µm

The application of the PAS staining method yielded satisfactory results in both paraffin- and JB-4-embedded liver sections (Fig. 4a, c, and e). In both techniques, cytoplasmic glycogen granules were clearly visualized. However, subtle morphological differences were noted: in paraffin sections, glycogen deposits appeared as finer, more delicate granules (Fig. 4a, c, and e), whereas in JB-4 sections, they often presented as coarser, more conspicuous aggregates within the cytoplasm (Fig. 4b, d, and f). Additionally, paraffin sections showed blue-stained nuclei due to Harris hematoxylin counterstaining, whereas nuclei remained unstained in JB-4 sections. This difference is attributable to the chemical properties of the embedding media: in paraffin, the hydrophobic matrix allows effective hematoxylin–DNA binding, while in JB-4, a hydrophilic glycol methacrylate resin, nuclear staining by hematoxylin is less efficient.

**Figure 5:**
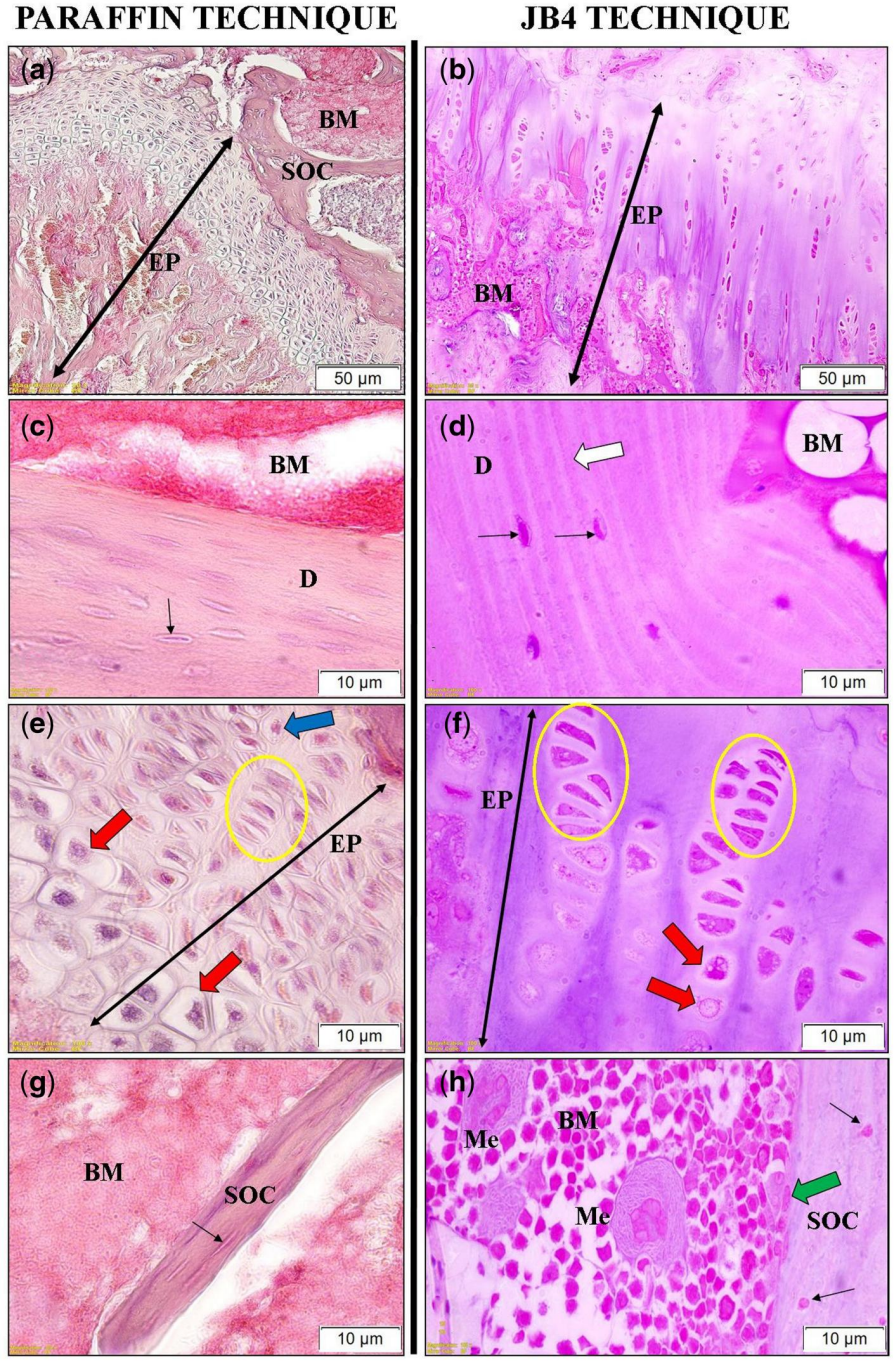
Morphological appearance of long bone sections using paraffin and JB-4 embedding techniques. EP: Epiphyseal plate; SOC: Secondary ossification center; BM: Bone marrow; D: Diaphysis; M: Meniscus; Me: Megakaryocytes; Purple arrow: Tendon of quadriceps femoris muscle; Double-headed black arrow: Epiphyseal plate region; Blue arrow: Chondrocytes in the resting zone; Yellow circled area: Chondrocytes in the proliferation zone; Red arrow: Chondrocytes in the hypertrophic zone; White arrow: Lamellar structures; Green arrow: Osteoblasts; Thin black arrow: Osteocytes. Scale bars: (**a**) and (**b**) = 50 µm; (**c**)–(**h**) = 10 µm. Panels (a), (c), (e), and (g) show hematoxylin&eosin (H&E) staining, while panels (b), (d), (f), and (h) show Acid Fuchsin&Toluidine Blue staining

In long bone samples, structural details were generally less distinct in paraffin-embedded sections. The lacunae of chondrocytes within the epiphyseal plate were more sharply defined in JB-4-embedded tissues (Fig. 5a, b, e, and f). Similarly, the nuclei of osteocytes embedded in the bone matrix, especially in the diaphysis (Fig. 5c and d) and secondary ossification zones (Fig. 5g and h), were more readily observed with the JB-4 technique. A particularly striking finding was the enhanced cytological detail in bone marrow cells (Fig. 5a–d, g), including clearly delineated demarcation membranes of megakaryocytes (Fig. 5h), observed exclusively in JB-4 preparations.

## Discussion

Histological techniques remain fundamental tools for examining the morphology of cellular and subcellular structures in tissue sections. Despite advancements in real-time imaging technologies, sectioning and staining procedures remain indispensable for visualizing cellular architecture within tissues. Depending on the experimental objective, numerous histological approaches—each offering advantages and drawbacks—may be employed. Among the most commonly applied methods are cryosectioning, paraffin embedding, and plastic resin embedding [7]. In this study, we compared the suitability of JB-4, a glycol methacrylate-based plastic resin, and conventional paraffin embedding for histological evaluation of liver and bone tissues. While paraffin embedding is widely utilized, our findings emphasize that selecting the appropriate embedding medium based on tissue type is crucial for achieving accurate histological interpretation.

For soft organs with high water content, such as the liver, paraffin embedding has traditionally been considered the standard technique due to its affordability, ease of application, compatibility with a wide range of stains, and generally good sectioning quality [4]. However, the high temperatures and harsh chemical solvents involved in the paraffin process can cause loss or degradation of specific cellular details [8]. In our study, paraffin-embedded liver tissues displayed adequate preservation of gross tissue architecture, though finer cytological features were less well-preserved. In contrast, JB-4-embedded sections preserved hepatocyte cords, central veins, sinusoidal spaces, and portal triad regions very well-defined. Moreover, intracellular components such as glycogen granules and Kupffer cell morphology were well-preserved, demonstrating JB-4’s effectiveness in maintaining fine tissue architecture.

Moreover, JB-4 permits polymerization at low temperatures, minimizing heat-related tissue damage and allowing for superior preservation of delicate structures [9]. Consistent with previous findings, JB-4 embedded tissues have been described as compatible with various staining methods, including histochemistry and, in some cases immunohistochemistry [10–12]. However, it is important to note that immunohistochemical methods were not applied in the present study, and further research is necessary to clarify the extent of compatibility with this technique. The water-soluble nature of JB-4 supports preservation of hydrophilic molecules and prevents the need for harsh clearing agents. Our observations showed enhanced cytological detail in JB-4 sections, particularly in the visualization of nuclear and cytoplasmic features, glycogen granules *via* PAS staining, and Kupffer cells. This improved clarity likely results from the water-compatible nature of glycol methacrylate, which minimizes the extraction of soluble carbohydrates during tissue processing. These results align with earlier reports that glycol methacrylate embedding facilitates the preservation of glycoconjugates [13, 14] and is effective for immunolocalization studies [15]. A concise comparison of key parameters between paraffin and JB-4 embedding techniques is summarized in Table 1.

**Table 1.** Comparison of key parameters between paraffin and JB-4 embedding techniques.

Parameter	Paraffin embedding	JB-4 embedding
Morphological preservation	Good for gross tissue architecture; fine cytological details partially lost	Excellent preservation of both tissue architecture and fine cytological details
Section thickness	4–6 µm (routine)	0.5–1 µm (ultrathin to semi-thin)
Staining compatibility	Compatible with wide range of stains, including H&E	Compatible with many stains; some limitations for routine H&E and IHC
Tissue type suitability	Soft tissues, widely used	Soft and mineralized tissues; small or delicate specimens
Equipment requirement	Standard microtome and routine lab setup	Specialized microtome and glass knives required
Cost	Low	Higher
Cytoplasmic detail	Moderate	High
Nuclear detail	Moderate	High

The superiority of JB-4 embedding was even more evident in the analysis of mineralized bone tissue. Decalcification, which is essential prior to paraffin embedding, often leads to structural distortion and loss of cytological detail [16]. In paraffin-embedded bone samples, features such as osteocyte lacunae, lamellae, and bone marrow elements were often poorly defined. In contrast, JB-4 sections revealed clear cellular morphology in bone tissues, including chondrocyte zones in the epiphyseal plate, osteoblasts, osteocytes, and the architecture of the bone matrix. Importantly, demarcation membranes of megakaryocytes in bone marrow were preserved and clearly visible—details absent in paraffin-embedded counterparts. These results corroborate the findings of Mulisch and Welsch [9], who highlighted its suitability for embryonic or small specimens due to its excellent infiltration properties.

A major advantage of JB-4 is its capacity to produce ultrathin (0.5–1 µm) or semi-thin (2–3 µm) sections, yielding cellular resolution approaching that of electron microscopy without requiring its complex instrumentation. However, this method also presents some limitations. It requires specialized equipment, such as microtomes compatible with hard plastic and glass knife-makers. In addition, while JB-4 works well with several stains, contrast may be suboptimal with routine hematoxylin-eosin staining compared to paraffin embedding, owing to differences in resin chemistry, tissue processing, and dye affinity. Additionally, JB-4 embedding typically incurs higher material costs [4, 5].

This study has several limitations. Firstly, the use of only five adult female *Wistar* albino rats as the experimental model limits the generalizability of the findings. Biological variability, including sex-related differences in liver and bone histology, may influence tissue preservation outcomes and should be considered in future studies. Increasing the sample size and including both sexes and different age groups would enhance the reliability of the results.

Only liver and long bone tissues were examined in this study; therefore, the comparative effectiveness and suitability of paraffin and JB-4 embedding techniques for other tissue types remain undetermined. Nevertheless, accumulating experience in our laboratory has demonstrated excellent preservation using JB-4 across a wide range of tissues, including pancreas, lung, skin, tongue, salivary gland, and lymph node (unpublished data). In addition, our recent comparative study on the female reproductive tissues of rats demonstrated highly satisfactory preservation using the JB-4 embedding technique [17]. Furthermore, in our doctoral research on human endometriosis tissue, JB-4 embedding provided excellent histological preservation, highlighting its potential applicability to human pathological specimens [18].

Advanced staining techniques such as immunohistochemistry were not applied, and thus the impact of the JB-4 technique on antigen preservation and detection was not directly evaluated. Previous studies have reported that while JB-4 preserves tissue morphology well, immunostaining outcomes can be limited. Proteolytic pre-treatment may increase antigen accessibility but can simultaneously cause ultrastructural damage, and colloidal gold-labeled antibodies often produce weaker signals compared to HRP-labeled antibodies, particularly at lower magnifications. These findings highlight potential limitations in antigen detection specifically when using JB-4 on prepared sections [7,19–21], and underscore the need for more careful optimization and comprehensive investigations.

JB-4 embedding method requires more specialized equipment and expertise, and it may be more costly compared to paraffin embedding, which could limit its practical use. Additionally, the inability to remove JB-4 resin from tissue sections may negatively affect some histochemical reactions and should be taken into consideration.

Finally, the study was limited to light microscopy analyses, without ultrastructural examinations. The use of electron microscopy or other advanced imaging techniques could provide more detailed cellular-level information. Considering these limitations, careful interpretation of the results is warranted, and future studies with broader scope and multidisciplinary approaches are recommended.

The choice between paraffin and JB-4 embedding techniques should be guided by the tissue type and the specific histological objectives of the analysis. Our results demonstrate that while paraffin embedding remains a practical and effective method for general examination of soft tissues like the liver, JB-4 provides superior preservation of cellular detail and structural integrity, especially in mineralized tissues such as bone. The capacity to produce thinner sections, combined with improved cytological resolution and compatibility with various staining techniques, makes JB-4 an advantageous alternative for detailed morpho-functional studies. These findings highlight the importance of methodological selection in histological research and suggest that JB-4 embedding should be considered when high-resolution morphological detail is essential.

## Data Availability

The datasets generated and analyzed during the current study are available from the corresponding author on reasonable request.
